# Characterization of the T cell receptor repertoire and melanoma tumor microenvironment upon combined treatment with ipilimumab and hTERT vaccination

**DOI:** 10.1186/s12967-022-03624-z

**Published:** 2022-09-11

**Authors:** Espen Basmo Ellingsen, Gergana Bounova, Iliana Kerzeli, Irantzu Anzar, Donjete Simnica, Elin Aamdal, Tormod Guren, Trevor Clancy, Artur Mezheyeuski, Else Marit Inderberg, Sara M. Mangsbo, Mascha Binder, Eivind Hovig, Gustav Gaudernack

**Affiliations:** 1 Department of Tumor Biology, Institute for Cancer Research, The Norwegian Radium Hospital, Oslo, Norway; 2 Faculty of Medicine, University of Oslo, Oslo, Norway; 3 Ultimovacs ASA, Oslo, Norway; 4 ENPICOM B.V., DA ’s-Hertogenbosch, Netherlands; 5 Department of Pharmacy, Science for Life Laboratory, Uppsala University, Uppsala, Sweden; 6 NEC Oncoimmunity, Oslo, Norway; 7 Department for Internal Medicine IV - Hematology and Oncology, Universitätsklinikum Halle (Saale), Halle, Germany; 8 Department of Oncology, Oslo University Hospital, Oslo, Norway; 9 HistoOne AB, Uppsala, Sweden; 10 Department of Cellular Therapy, Oslo University Hospital, Oslo, Norway; 11 Ultimovacs AB, Uppsala, Sweden; 12 Centre for Bioinformatics, University of Oslo, Oslo, Norway

**Keywords:** Cancer, Immunotherapy, Therapeutic Cancer Vaccine, Telomerase, hTERT, Melanoma, Ipilimumab

## Abstract

**Background:**

This clinical trial evaluated a novel telomerase-targeting therapeutic cancer vaccine, UV1, in combination with ipilimumab, in patients with metastatic melanoma. Translational research was conducted on patient-derived blood and tissue samples with the goal of elucidating the effects of treatment on the T cell receptor repertoire and tumor microenvironment.

**Methods:**

The trial was an open-label, single-center phase I/IIa study. Eligible patients had unresectable metastatic melanoma. Patients received up to 9 UV1 vaccinations and four ipilimumab infusions. Clinical responses were assessed according to RECIST 1.1. Patients were followed up for progression-free survival (PFS) and overall survival (OS). Whole-exome and RNA sequencing, and multiplex immunofluorescence were performed on the biopsies. T cell receptor (TCR) sequencing was performed on the peripheral blood and tumor tissues.

**Results:**

Twelve patients were enrolled in the study. Vaccine-specific immune responses were detected in 91% of evaluable patients. Clinical responses were observed in four patients. The mPFS was 6.7 months, and the mOS was 66.3 months. There was no association between baseline tumor mutational burden, neoantigen load, IFN-γ gene signature, tumor-infiltrating lymphocytes, and response to therapy. Tumor telomerase expression was confirmed in all available biopsies. Vaccine-enriched TCR clones were detected in blood and biopsy, and an increase in the tumor IFN-γ gene signature was detected in clinically responding patients.

**Conclusion:**

Clinical responses were observed irrespective of established predictive biomarkers for checkpoint inhibitor efficacy, indicating an added benefit of the vaccine-induced T cells. The clinical and immunological read-out warrants further investigation of UV1 in combination with checkpoint inhibitors.

*Trial registration* Clinicaltrials.gov identifier: NCT02275416. Registered October 27, 2014. https://clinicaltrials.gov/ct2/show/NCT02275416?term=uv1&draw=2&rank=6

**Supplementary Information:**

The online version contains supplementary material available at 10.1186/s12967-022-03624-z.

## Background

T cells that recognize tumor antigens are the foundation of current immunotherapy, on which the efficacy of checkpoint inhibitors (CPIs) relies. CPIs have revolutionized the treatment of many cancers, most notably malignant melanomas [1, 2]. Although many patients experience deep and durable clinical responses to CPIs, unfortunately, many patients progress and require additional treatment. CPIs work by inhibiting axes that restrict T cell activation and proliferation, releasing spontaneously primed antitumor immune responses [3]. Therefore, predictive biomarkers for CPI efficacy are largely related to tumor immunogenicity, such as tumor mutational burden [4], neoantigen burden [5], PD-L1 positivity [6], tumor-infiltrating lymphocytes [7], and IFN-γ gene signature [8]. Conversely, a lack of CPI efficacy is associated with inadequate antitumor T cell responses and immunosuppressive factors in the tumor microenvironment (TME) [9]. Although incremental benefits are achieved by simultaneously inhibiting multiple immune checkpoints [10], the quality and quantity of T cells specific for tumor antigens remains a limiting factor for further advancements in immunotherapy.

Therapeutic cancer vaccines (TCVs) aim to direct the expansion of T cells targeting relevant tumor antigens, providing a new wave of tumor-specific T cells to the TME [11]. This approach thereby serves to supplement and reinforce the anti-tumor immune response, synergizing with immunotherapies that depend on the presence of tumor-specific T cell responses (e.g. CPIs). Strategies applied to TCVs include targeting neoantigens and shared tumor-associated antigens (TAAs). Tumor-specific somatic mutations may give rise to aberrant peptides that are sufficiently different from their normal counterparts, allowing recognition of these neoantigens by the patient’s T cells. In contrast, TAAs are non-mutated antigens with a selective expression pattern that is preferentially limited to tumors. Personalized neoantigen cancer vaccines require tumor tissue harvesting and next-generation sequencing for in silico neoantigen prediction, and subsequent personalized vaccine production. TAA-based vaccines, however, can forego personalized production and can be delivered directly off-the-shelf to the patient, assuming that the tumor expresses the target antigen. TAA-based vaccines may also be more relevant in cancers with low tumor mutational burden, where fewer neoantigens are presented.

Telomerase reverse transcriptase (hTERT) is a TAA activated in 85–90% of all tumor types [12, 13]. Telomerase is expressed by cancer cells to maintain telomere replication supporting unconstrained cancer cell proliferation and metastasis [14, 15]. Therefore, telomerase activation is considered a hallmark of cancer [16]. Melanomas frequently harbor hTERT promoter mutations and gene copy number amplification which are associated with increased hTERT expression [17–19]. These genomic aberrations collectively lead to an increased antigen presence providing a scientific rationale for targeting hTERT in melanomas. High tumor telomerase activity is a well-established negative prognostic factor across multiple cancer indications [20–24], whereas anti-telomerase CD4 T cell immune responses are emerging as independent positive prognostic factors validated in several malignancies [25–27]. Based on these characteristics, hTERT is considered a promising TAA for therapeutic vaccination [28].

UV1 is a TCV composed of three synthetic long peptides derived from the active site of hTERT and has been proven to establish robust, long-lasting T cell responses across an HLA-unselected population in three completed phase I clinical trials [29]. Immune responses induced by UV1 have been identified as CD4 Th1-polarized effector memory cells with inflammatory cytokine profiles (tumor necrosis factor-α and IFN-γ). Expanding a population of CD4 Th1 cells targeting a shared tumor antigen could lead to intratumoral re-activation of these cells, inducing an inflammatory TME and immune-mediated cancer cell death [30–32]. CD4 T cells enhance antitumor immunity by licensing dendritic cells for effective antigen presentation and by secretion of inflammatory cytokines, promoting immune cell infiltration, and effector functions.

Immune checkpoints maintain immune responses within a desired physiological spectrum, and their blocking is expected not only to disinhibit spontaneously primed anti-tumor T cell responses, but also de novo T cell responses induced by vaccination. This provides a therapeutic rationale for combining checkpoint inhibition with TCVs. The cytotoxic T lymphocyte-associated protein 4 (CTLA-4) immune checkpoint competitively inhibits the binding of CD28 on T cells with CD80/CD86 on antigen-presenting cells, thereby reducing T cell activation by preventing the co-stimulation of primed T cells. Ipilimumab is a monoclonal antibody that blocks this immune checkpoint and disrupts negative regulation imposed by CTLA-4. We wanted to explore whether combining ipilimumab with the UV1 vaccine would lead to synergy in terms of expanding vaccine-specific T cells, improving anti-tumor immune responses, and clinical outcomes.

We have previously reported safety and feasibility data of this phase I/IIa clinical trial evaluating combined UV1 vaccination and ipilimumab in patients with metastatic melanoma [33]. A parallel phase 4 trial evaluating ipilimumab monotherapy at Norwegian hospitals during the same period has since been published, demonstrating clinical outcomes aligned with previously reported data on ipilimumab monotherapy [34, 35]. Considering the combination study yielded comparatively superior progression-free survival, overall survival, and objective responses rate, we sought to further investigate whether this cohort comprised patients with favorable baseline characteristics and explore the dynamics of the vaccine-induced immune response. Herein, we report an updated survival analysis and extensive translational research from this clinical trial.

## Methods

### Study design, patients, and treatments

The study design, eligibility criteria, and treatments have been previously described [33]. The UV1/hTERT-MM study was an open-label, single-arm, single-center, phase I/IIa clinical trial (NCT02275416). The primary objective of this study was to assess the safety of ipilimumab combined with UV1 vaccination in patients with malignant melanoma. The secondary objectives included immune response assessment, objective response rate (ORR) per RECIST 1.1, overall survival (OS), and progression-free survival (PFS). Key eligibility criteria were age ≥ 18 years and a histologically confirmed diagnosis of unresectable stage III/IV cutaneous malignant melanoma. An Eastern Cooperative Oncology Group (ECOG) performance status ≤ 1 and any previous therapies for melanoma were permitted. The study participants provided written informed consent prior to enrolment. The study was approved by the competent regulatory authority and independent ethics committee.

UV1 consists of three synthetic long peptides derived from the active site of telomerase reverse transcriptase (hTERT 660–689 termed p719-20; hTERT 691–705 termed p725; hTERT 651–665 termed p728). A total of 300 µg of lyophilized peptides in equimolar amounts was reconstituted in water for injection and administered intradermally to the lower abdomen. Granulocyte–macrophage colony-stimulating factor (GM-CSF, sargramostim) (Leukine, Sanofi Aventis, Bridgewater, NJ, US) was used as a vaccine adjuvant at 75 µg and was injected intradermally at the same site 10–15 min before UV1. Ipilimumab (3 mg/kg) was administered according to the label for up to 4 infusions. Patients received up to nine UV1 vaccinations, initiated one week prior to the first dose of ipilimumab.

### Immune response assessment

Peripheral blood mononuclear cells (PBMCs) were isolated from whole blood samples (50 ml in acid dextrose tubes) at baseline and at frequent intervals during the treatment and long-term follow-up periods, as previously described [29]. Briefly, vaccine-specific T cell immune responses were assessed using a standard proliferation assay (^3^H-Thymidine incorporation). PBMCs were pre-stimulated in vitro for 10–12 days with a mixture of vaccine peptides at 10 μM. After pre-stimulation, the cells were re-stimulated for 48 h with or without 10 μM vaccine peptides using irradiated autologous PBMCs as antigen-presenting cells, and tested in triplicate for proliferation by ^3^H-thymidine incorporation. The stimulation index (SI) was calculated by dividing the mean proliferation count in vaccine peptide-stimulated wells by the mean proliferation count in unstimulated wells. A three-fold increase in proliferation towards any of the three peptides, or a mixture of these, was considered an immune response-positive sample. Staphylococcus aureus enterotoxin C3 (SEC3) was used as the positive control in the immune response assay. The reported SI values were those observed during the treatment period, defined as up to 16 weeks after the last vaccination.

### TCR sequencing

DNA was extracted from PBMC samples of all patients at baseline and up to two time points thereafter using the GenElute Mammalian Genomic DNA Kit (Sigma Aldrich), according to the manufacturer´s instructions (Additional file 1: Table S1). In addition, DNA extraction was performed on stimulated PBMC samples after a 10-day in vitro stimulation (N01, N02, N07, and N09) and biopsies from patients N02 and N03.

The T cell receptor beta (TRB) locus was amplified from up to 250 ng of genomic DNA, as described previously [36, 37]. Briefly, a TRB repertoire library was generated using two consecutive polymerase chain reactions. First, the rearranged TRB locus was amplified and sample-specific barcodes were added to the amplicons. Library concentrations and sizes were determined using a Qubit (Thermo Fisher) and Bioanalyzer (Agilent), respectively. The final library was sequenced on an Illumina MiSeq with the MiSeq Reagent Kit v3 (600-cycles) chemistry.

### TCR profiling and processing

Sequencing quality was assessed before and after repertoire sequencing data processing with IGX Inspect (IGX Platform 3.0.6 August 2021), a quality control application designed for immune receptor sequencing data. Checks included standard fastq quality metrics, such as average read quality, Q30 scores, as well as V and J gene alignment distributions and read fate, and a percentage breakdown of the receptor extraction status of raw reads. All the samples had good sequence quality and a high percentage of reads with successfully extracted receptors (all > 95%, except for one sample with 90% and one with 70%).

Raw fastq files were processed using the IGX Profile (IGX Platform 3.0.6 August 2021), a tool that parses immune receptor structural components by aligning germline genes and adaptively correcting errors based on the overall sample quality. The IGX Profile provided receptor annotation with complementarity-determining region 3 (CDR3) sequences, V and J gene assignments, functionality, alignment scores, and quality information. All receptors with the same CDR3 amino acid sequence were considered instances of the same clone and all analyses were performed at the clone level.

Count normalization of TCR repertoires was performed by downsampling. Identification of significantly expanded clones was performed using EdgeR [38]. The expanded clones already present in the baseline sample were filtered out. A more detailed description of these methods is provided in the Additional file 1.

### Multiplex immunofluorescence staining

Biopsies were harvested at baseline from nine patients and at week 12–15 from five patients (Additional file 1: Table S2). One part of the biopsy was snap-frozen in liquid nitrogen and stored at − 80 °C, whereas the other was formalin-fixed and paraffin-embedded (FFPE). FFPE biopsies were used for multiplex immunofluorescence staining. Biopsy Sects. (4 µm thick) were stained using a custom-based 5-color IHC kit (Akoya Biosciences, Marlborough, MA, USA) and the fully automated Leica Bond RXm (Leica Biosystems, Buffalo Grove, IL, USA). The slides were deparaffinized, rehydrated, and rinsed with distilled H_2_O. Antigen retrieval and removal of antibodies from the previous cycles were performed by boiling at 95 °C at pH 9 (first cycle) or pH 6 (all remaining cycles).

For multiplex immunofluorescence staining, a panel of immune markers was developed using antibodies against CD4 (rabbit/ERP6855, Abcam, 1:80), CD8α (mouse/144B, Invitrogen/MA5-13,473, 1:100), PD-L1 (rabbit/E1L3N, Akoya, ready to use), and TERT (rabbit/ab230527, Abcam, 1:400). A cocktail of two antibodies was used to identify the melanoma cells: anti-Sox10 (rabbit/EP268-1, Akoya, ready to use) and anti-S100 (mouse/4C4.9, Akoya, ready to use). Staining was developed using amplification HRP-polymer systems and Opal fluorophore dyes (see Additional file 1: Table S3). To visualize the cell nuclei, the tissue was stained with 4′,6-diamidino-2-phenylindole (Spectral DAPI, Akoya). The slides were mounted with Prolong Diamond Antifade Mountant (Thermo Fisher, Waltham, MA, USA) and imaged at × 20 magnification using the Vectra® Polaris™ Automated Quantitative Pathology Imaging System (Akoya Biosciences, Marlborough, MA, USA). Each image was manually reviewed and curated by a pathologist to exclude artifacts and staining defects.

### Whole-exome and RNA sequencing and downstream analyses

Snap-frozen biopsies were disrupted on a TissueLyser LT, followed by DNA extraction using the AllPrep DNA/RNA/miRNA Universal Kit (Qiagen, Hilden, Germany). RNA extraction was performed using a GenElute™ Total RNA Purification Kit (Merck). Biopsy DNA and RNA extracts were obtained from nine patients at baseline and five at week 12–15.

Whole-exome sequencing (WES) was performed as previously described in Aamdal et al. [33]. Briefly, 1 µg of DNA was used as the starting material for exome library preparation using the Agilent AllExome V5 kit, according to the manufacturer’s protocol. Sequencing was performed pair-ended, generating approximately 90 M PE reads per tumor and 40 M PE reads per normal, using sequencing by synthesis chemistry on a HiSeq4000 system. Variant calling was performed as previously described [33]. Tumor mutational burden (TMB) was defined as the number of non-synonymous variants with an allelic frequency of > 5% per megabase.

RNA samples were processed using an Illumina TruSeq stranded mRNA kit with 100 ng as the starting material. RNA sequencing was performed on the NextSeq500 using two HighOutput flow cells with 75 bp single-read sequencing. Hierarchical clustering of the genes included in the IFN-γ signature [8] was performed using Euclidean distance with the Morpheus tool (https://software.broadinstitute.org/morpheus). HLA class I expression was assessed for HLA-A, -B, and -C, and HLA-DP, -DQ, and -DR for class II, as previously described [39]. Differentially expressed genes post-treatment compared to baseline were assessed using the NOISeq tool [40] and were calculated for each patient with available biopsies (N01, N02, N03, and N07). Gene set enrichment analysis of the differentially expressed genes was performed using WebGestalt [41] and Gene Ontology mapping to the Biological Processes functional database.

The artificial intelligence (AI) prediction platform used for immunogenic neoantigen prediction was the NEC Immune Profiler (NIP) [42]. The NIP software predicted each of the key determinants of antigen presentation (AP) for each somatic mutation, by predicting the potential of all tumor-specific mutated peptides to be efficiently presented by each of the patients Class I HLA-A and -B alleles.

### Statistics

The sample size (n) represents the number of patients or samples analyzed. Survival analyses were performed using the Kaplan–Meier method. All statistical analyses were performed using GraphPad Prism version 9.2.0. (GraphPad Software). Statistical significance was set at *p* < 0.05.

## Results

### Clinical outcome of combined ipilimumab and UV1 treatment

**Fig. 1:**
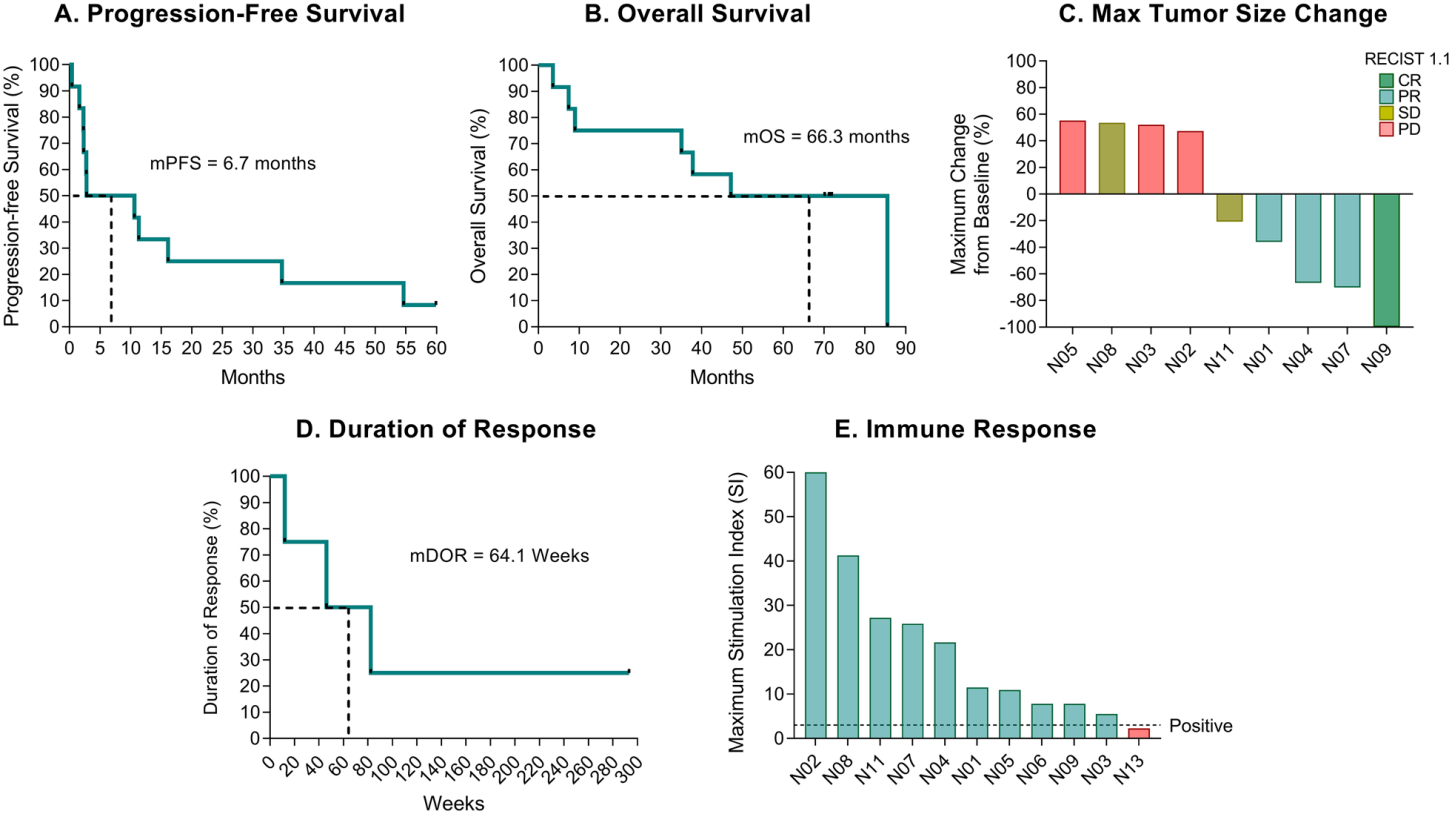
Treatment outcomes and anti-hTERT immune response induction in patients treated with UV1 and ipilimumab. **A** Progression-free survival and **B** overall survival in all patients enrolled (n = 12). **C** Maximum change in the sum of longest diameter in RECIST 1.1 evaluable patients (n = 9). **D** Duration of response (n = 4). **E** Maximum T cell proliferation response in terms of stimulation index (SI) in evaluable patients (n = 11). The dotted line represents positivity threshold (SI ≥ 3). *CR* complete response, *PR* partial response, *SD* stable disease, *PD* progressive disease, *IR* immune response

We have previously presented patient demographics and 5-year clinical follow-up data [33]. Between January and October 2015, 12 patients with stage IV melanoma were enrolled in this study. Patients received a mean of 5.5 UV1 vaccinations (range 3–9) and 3.2 courses of ipilimumab (range 1–4). UV1 was considered safe and well-tolerated, with most adverse events being grade 1–2 injection site reactions. With up to 85.5 months of clinical follow-up, the median PFS was 6.7 months (Fig. 1A), and the median OS was 66.3 months (Fig. 1B). The ORR was 33% (three partial responses and one complete response) (Fig. 1C). The median time to clinical response was 30.2 weeks (range 16.4–155.3), and the median duration of response was 64.1 weeks (range 12-not reached) (Fig. 1D). One patient (N09) had a sustained complete response lasting more than 5.5 years. A vaccine-induced anti-UV1 immune response was demonstrated in 10/11 evaluable patients (91%). The median of maximum in vitro T cell proliferation response (stimulation index) across all patients was 11.5 (range 2.3–60.0). Patient N02 exhibited the strongest immune response, with a 60-fold increase in T cell proliferation response to in vitro vaccine peptide stimulation (Fig. 1E).

### Baseline tumor microenvironment and TCR repertoire status

**Fig. 2:**
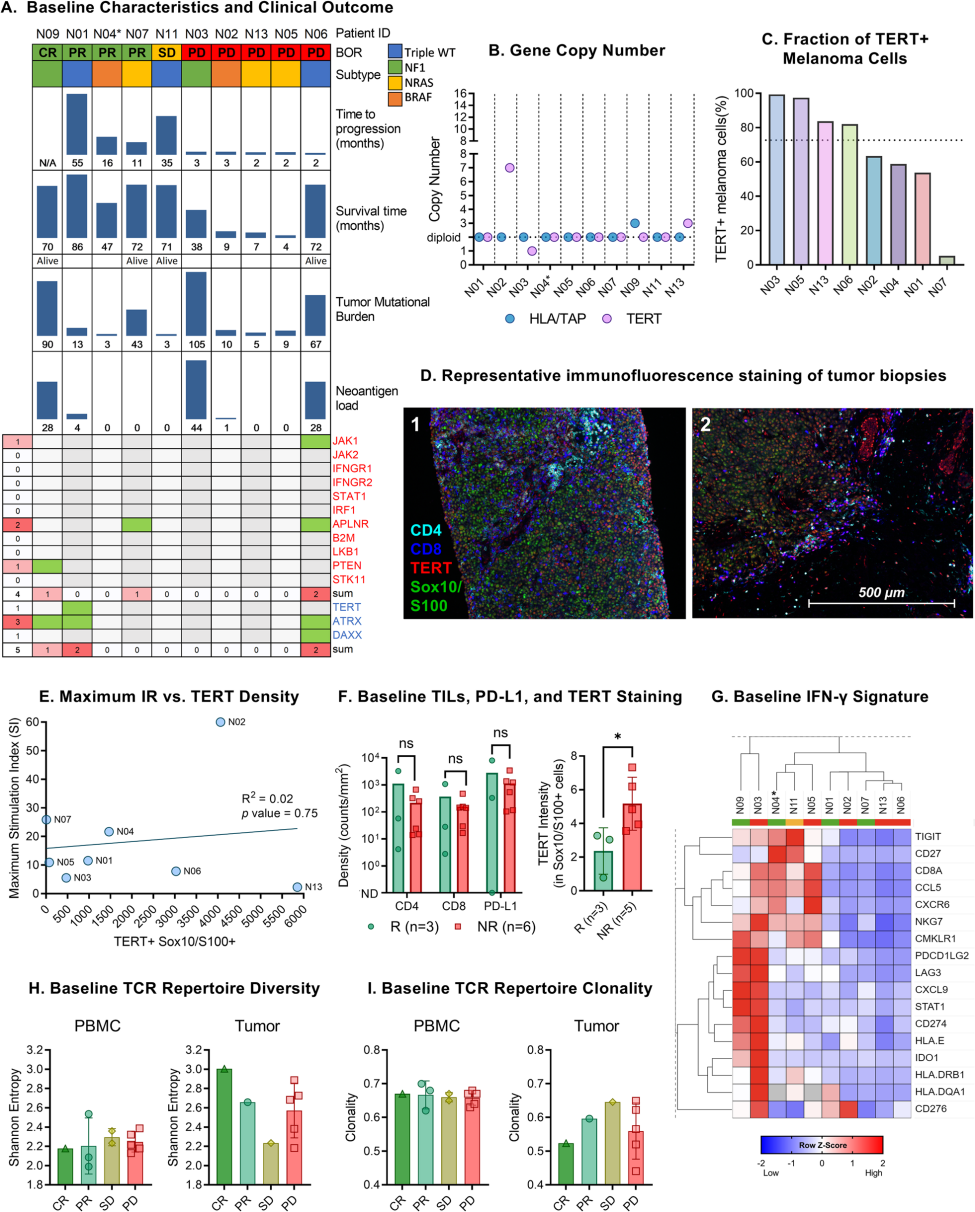
Baseline tumor microenvironment. **A** Clinical outcome and genomic characteristics of patients with available biopsies at baseline. Genes in red font are related to immunotherapy resistance [45], and genes in blue telomerase expression. Green squares indicate non-synonymous mutations. **B** Gene copy alterations of TERT, HLA, and TAP. **C** Fraction of Sox10/S100 cells also positive for hTERT based on immunofluorescence staining (dotted line represents the median, 72.7%). **D** Representative immunofluorescence staining of tumor biopsies. CD4 is stained cyan, CD8 is stained blue, hTERT is stained red, and Sox10/S100 is stained green. Image 1 is from patient N04 at baseline, and image 2 from patient N06 at baseline. The hTERT dense area in the top right of image 2 is likely a hair follicle. **E** Linear regression analysis of immune response (maximum stimulation index (SI)) and hTERT + Sox10/S100 + cell density based on immunofluorescence staining. The association is not significant (Pearson’s correlation, *p* = 0.47). **F** Baseline CD4, CD8, and PD-L1 density in responders (R) and non-responders (NR) (Mann–Whitney test, CD4 *p* = 1.0; CD8 *p* = 0.71; PD-L1 *p* = 0.90). Baseline hTERT intensity in melanoma cells was significantly lower in clinical responders (Mann–Whitney test, *p* = 0.04). **G** Hierarchical clustering of baseline IFN-γ signature using the Euclidean distance. The grey box indicates missing data (HLA-DQA1 only). **H** Baseline PBMC and tumor TCR repertoire diversity and **I** clonality according to RECIST 1.1 response categories. BOR, best overall response; ns, not significant; ND, not detected. *Patient N04 only had week 12 biopsy available for whole-exome and RNA sequencing. The BRAF status was based on a diagnostic biopsy for this patient

There was no apparent association between baseline tumor mutational burden (TMB) or neoantigen load and response to therapy (Fig. 2A). One patient had a missense mutation in hTERT (*Glu429Lys*). However, the mutation did not map to epitopes corresponding to UV1 peptides and did not persist in the post-treatment biopsy (Additional file 1: Figure S1). Alternative lengthening of telomeres (ALT) has been described as an hTERT-independent telomere maintenance mechanism that may limit the efficacy of hTERT-targeting T cells. Therefore, we examined the presence of loss-of-function mutations in two genes, ATRX and DAXX, known to induce the ALT phenotype [43]. No nonsense or frameshift mutations were detected; however, we observed missense mutations in ATRX in patients N09 (*Met352Ile*) and N01 (*Glu2105Lys*), and both ATRX and DAXX mutations in patient N06 (*Asp2048Asn*, *Ser377Phe*). The ATRX mutation present at baseline in patient N01 was not detected in the biopsy specimen harvested at week 12 (Additional file 1: Figure S1).

Two patients had tumors polyploid of the hTERT gene. Patient N02 had seven copies of hTERT at baseline, whereas patient N13 had three copies (Fig. 2B). We further investigated copy number alterations in HLA and TAP genes, as their loss have been described as a mechanism of resistance to immunotherapy [44]. Most patients were diploid for HLA and TAP, and we did not observe any loss of heterozygosity for these genes (Fig. 2B).

Melanoma cell hTERT expression was determined by co-staining for Sox10/S100 and hTERT and was confirmed in all evaluable biopsies (baseline sample from patient N11 was not evaluable because of a lack of Sox10/S100 stained cells). The median fraction of hTERT-positive melanoma cells was 72.7% (range 5.2–99.3) (Fig. 2C). Two patients (N02 and N13) that were polyploid for the hTERT gene also had the highest hTERT + Sox10/S100 density (Fig. 2E). Interestingly, patient N02 displayed the highest in vitro T cell proliferation response against UV1 peptides, but patient N13 did not demonstrate a vaccine-specific T cell response. However, patient N13 had only one post-vaccination sample for the immune response assessment at week 4. There was no overall association between tumor hTERT density and vaccine-specific peripheral T cell proliferation response (Fig. 2E). Baseline PD-L1 expression and infiltration of CD4 or CD8 T cells were not significantly different between responders and non-responders (Fig. 2F). However, the baseline hTERT intensity in Sox10/S100 positive cells was significantly lower in the clinical responders (Fig. 2F).

We performed RNA sequencing of the available biopsies and found no apparent association between the baseline IFN-γ gene signature and response to therapy (Fig. 2G). Patient N06, with a JAK1 mutation, demonstrated relatively low expression of the IFN-γ signature. Baseline HLA class I and II expression levels were not significantly different between the clinical responders and non-responders (Additional file 1: Figure S2).

Across the clinical response categories, we observed a trend towards an inverse relationship between baseline PBMC and biopsy T cell receptor (TCR) repertoire diversity. The complete response patient (N09) had the highest intratumoral TCR diversity at baseline, and comparatively low PBMC diversity. The diversity and clonality of PBMC samples at baseline were fairly even across clinical response categories (Fig. 2H and I).

### Evolution of the TCR repertoire and tracking of vaccine-enriched clonotypes

**Fig. 3:**
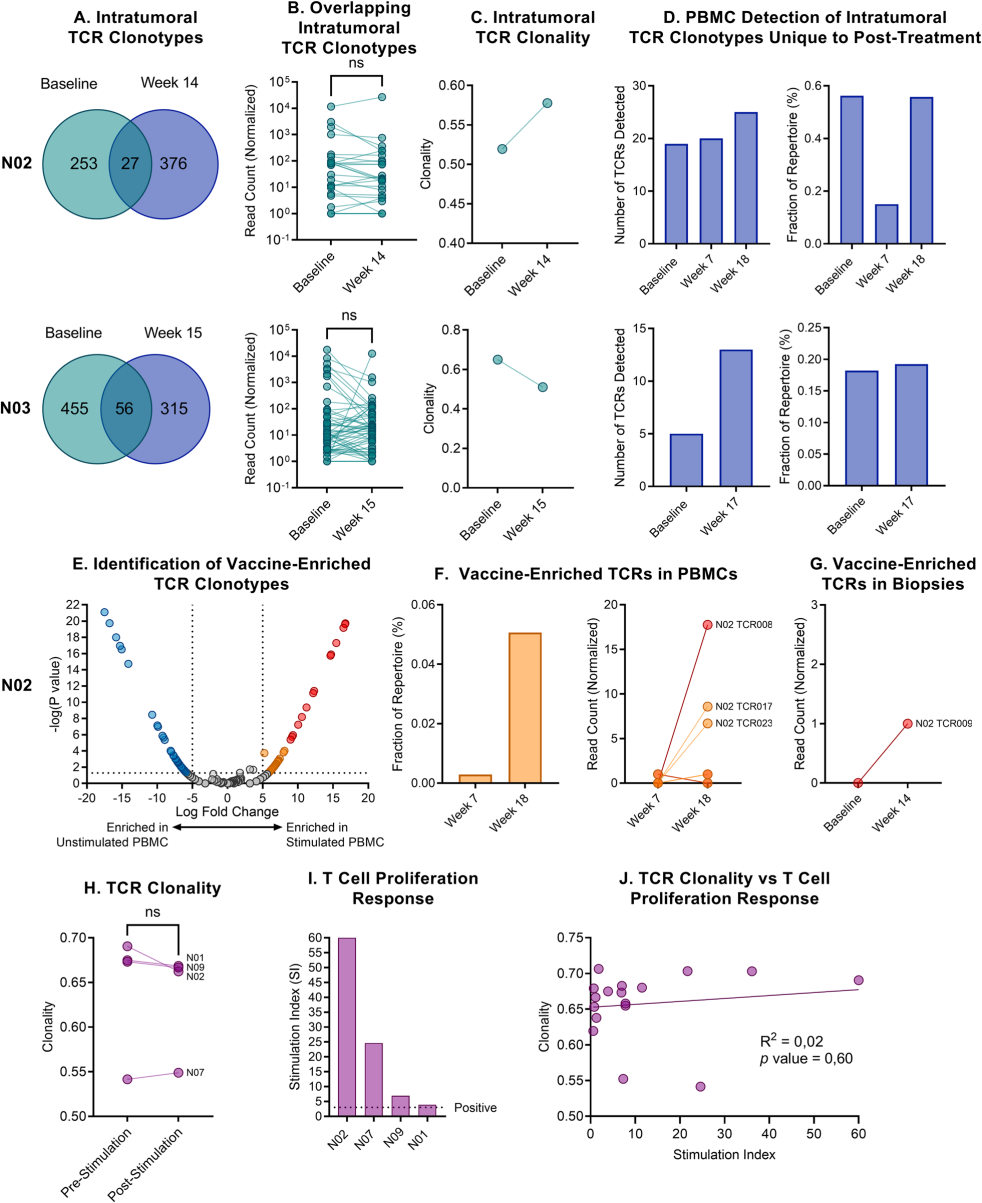
Evolution of the TCR repertoire on treatment. **A** Overlapping intratumoral TCR clonotypes at baseline and post-treatment for patient N02 and N03. **B** Relative abundance (normalized read count) for persisting intratumoral TCRs between baseline and post-treatment. **C** Intratumoral TCR clonality at baseline and post-treatment. **D** The number of clonotypes unique to the post-treatment biopsy also detected in PBMC samples, and their fraction of the TCR repertoire. The TCR repertoire fraction was calculated by summing the normalized read count for each TCR and dividing by the total read count for the same sample. **E** Volcano plot illustrating enriched TCRs after a 10-day in vitro peptide stimulation of PBMCs. Orange dots indicate TCRs with a log fold change above 5, unadjusted *p* < 0.05, and red dots adjusted *p* < 0.05. **F** Vaccine-enriched TCRs identified in unstimulated PBMC samples. TCR clonotypes are labeled according to their rank in terms of log fold change after stimulation. **G** Vaccine-enriched TCRs identified in tumor biopsies. **H** Sample clonality pre and post-10-day in vitro vaccine peptide stimulation. **I** T cell proliferation responses from the same samples. **J** Unstimulated PBMC sample clonality vs. in vitro T cell proliferation response

We matched the baseline and post-treatment (week 14–15) tumor biopsy TCR sequencing data available from two patients (N02 and N03, both with PD as BOR). Most intratumoral TCR clonotypes changed between baseline and post-treatment (Fig. 3A). The overlapping TCRs did not have significantly different relative abundances after treatment, indicating that treatment did not lead to intratumoral expansion of the T cells (Fig. 3B). Although there was an increase in total unique TCR clonotypes post-treatment for patient N02, intratumoral TCR clonality increased (Fig. 3C), indicating that fewer TCR clonotypes constituted a larger proportion of the intratumoral TCR repertoire. The opposite was observed for patient N03. We investigated the PBMC TCR repertoire for TCRs unique to the post-treatment biopsies, observing an increasing number of these clonotypes upon treatment, indicating peripheral expansion of these clonotypes. However, these TCRs did not constitute a considerably larger fraction of the total TCR repertoire over time (Fig. 3D).

From week 7 PBMC samples, TCR sequencing was performed before and after 10 days in vitro stimulation with the vaccine peptides in patients N01, N02, N07, and N09. We assessed whether vaccine-enriched TCR clonotypes were detectable in unstimulated PBMCs and a biopsy at later time points (only patient N02 had available calculated vaccine-enriched TCRs and a post-treatment biopsy) (Fig. 3E). In patient N02, we observed three clones expanding from week 7 to week 18 (Fig. 3F), and a single vaccine-enriched TCR clone was detected in the biopsy post-treatment (Fig. 3G). For patients N01, N07, and N09, we observed similar findings, with single clones expanded in later PBMC samples, reaching up to 12% of the entire repertoire (Additional file 1: Figure S3).

To assess whether in vitro stimulation induced the expansion of a few single T cell clones, we compared the TCR clonality of the unstimulated and stimulated PBMC samples (Fig. 3H) and observed no trend towards increased clonality despite strong T cell proliferation responses in the same sample, especially evident in patient N02 (Fig. 3I). Furthermore, there was no overall correlation between in vitro T cell proliferation responses and TCR clonality in the unstimulated samples (Fig. 3J).

### Evolution of the tumor microenvironment

**Fig. 4:**
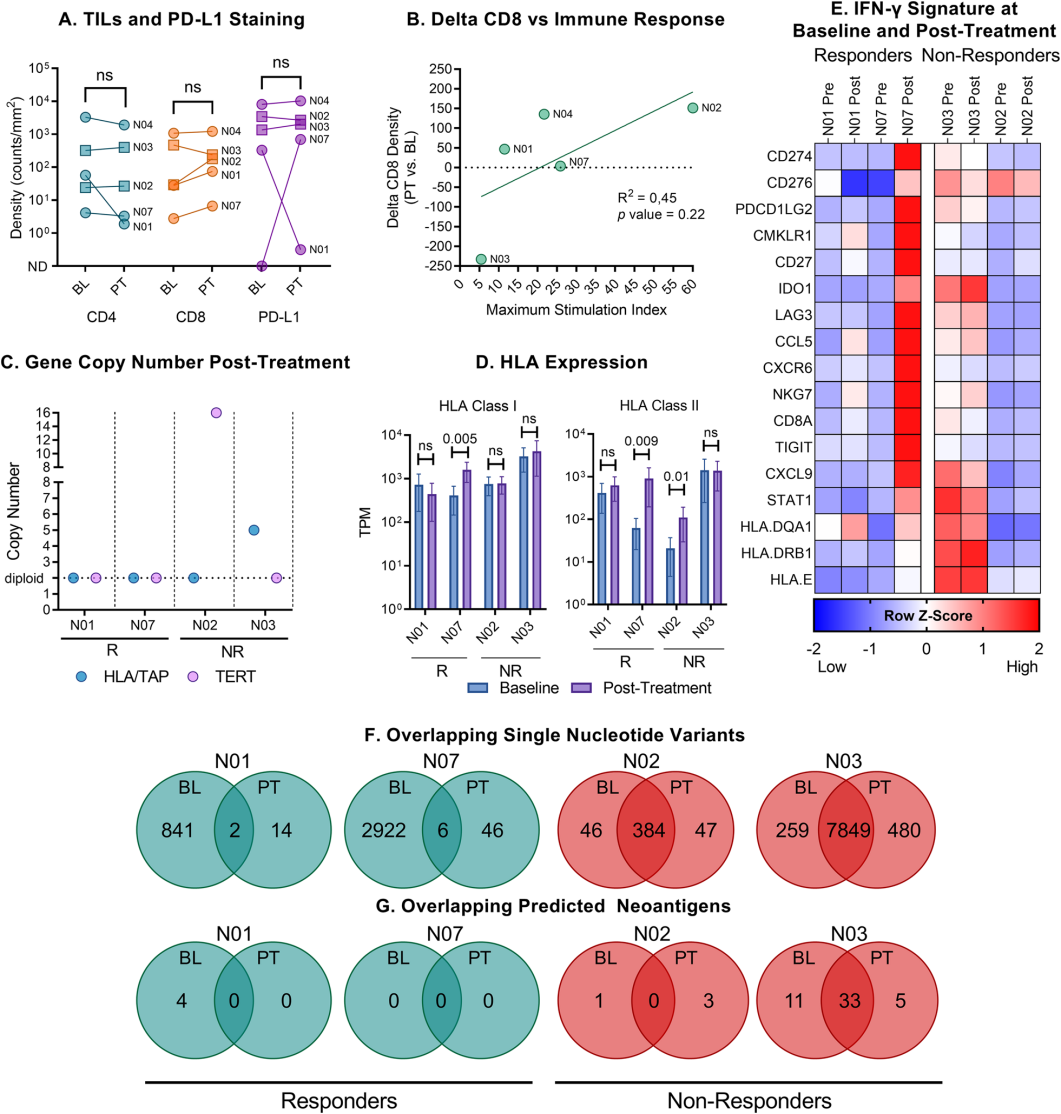
Evolution of the tumor microenvironment on treatment. **A** CD4, CD8, and PD-L1 density in baseline and post-treatment biopsies. Circles indicate clinical responders and squares non-responders. The difference between baseline and post-treatment was not significant (Mann–Whitney test, CD4 *p* = 0.69; CD8 *p* = 0.69; PD-L1 *p* = 0.84). **B** Linear regression analysis of difference in CD8 density between post-treatment and baseline versus maximum T cell proliferation response (Pearson’s correlation, *p* = 0.22). **C** Copy number status of HLA/TAP and hTERT genes post-treatment. **D** HLA class I and II expression in baseline and post-treatment biopsies. The *p* values represent unpaired t test for difference between baseline and post-treatment expression levels. **E** IFN-γ signature in baseline vs. post-treatment biopsies. **F** Overlapping single nucleotide variants and **G** predicted neoantigens between baseline and post-treatment biopsies. *IR* immune response, *TIL* tumor-infiltrating lymphocytes, *R* clinical responder, *NR* clinical non-responder, *BL* baseline, *PT* post-treatment, *NS* not significant, *TPM* transcripts per million, *ND* not detected

There was no significant overall increase in tumor-infiltrating lymphocytes (TILs) or PD-L1 expression in the five patients with available baseline and post-treatment (week 12–15) biopsy immunofluorescence staining. An increase in CD8 density was observed in patients N01, N02, and N07 (Fig. 4A), and we observed a non-significant trend towards increased delta CD8 density with increased vaccine-specific peripheral T cell responses (Fig. 4B).

No loss of heterozygosity for either the HLA/TAP or hTERT genes was observed in post-treatment biopsies (Fig. 4C). A relative increase in the expression of both HLA class I and II genes was observed in patient N07 post-treatment, and class II only in patient N02 (Fig. 4D). For the genes included in the IFN-γ signature, we observed a relative increase in expression post-treatment in responding patient N07, and to a lesser degree in patient N01 (Fig. 4E). The two progressors did not show considerable changes in IFN-γ signature upon treatment. A similar expression pattern was observed for genes related to T cell function and activation, immune checkpoint molecules, and cytokine activity (Additional file 1: Figure S4).

We evaluated mutational contraction and expansion in four patients with available baseline and post-treatment biopsy whole-exome sequencing data. The responding patients (N07 and N01) exhibited a drastic reduction in total single nucleotide variants (SNVs), with only a few overlapping between the two time points. Conversely, the two progressors had a high degree of overlapping SNVs, indicating a limited impact of the treatment and negligible killing of cancer cells harboring these mutations (Fig. 4F). The four neoantigens present at baseline in responding patient N01 were not observed post-treatment, whereas most neoantigens persisted in non-responding patient N03 (Fig. 4G).

Gene set enrichment analysis was performed on differentially expressed genes post-treatment for the four patients with matching biopsies, observing enriched gene sets related to immune responses in the responding patients, such as “T cell activation”, “Leukocyte proliferation”, although with an FDR > 0.05 (Additional file 1: Figure S5). The two progressors did not show enrichment in gene sets related to adaptive immune responses.

## Discussion

The anti-CTLA-4 monoclonal antibody ipilimumab was the first checkpoint inhibitor (CPI) to receive Food and Drug Administration approval for the treatment of metastatic malignant melanoma. Here, we report translational research and updated clinical follow-up of 12 patients with malignant melanoma enrolled in a clinical trial evaluating ipilimumab and the TCV candidate UV1. Since the completion of this clinical trial, PD-1 inhibitors, either as single agents or in combination with a CTLA-4 or a LAG-3 inhibitor, have replaced ipilimumab monotherapy as the standard of care for metastatic melanoma. While ipilimumab led to a median OS of approximately 10 months [35], the combination of nivolumab and ipilimumab further improved clinical outcomes, exhibiting a 6-year overall survival rate of approximately 50% [2]. Despite these advancements, insufficient T cell responses remain a limiting factor for the efficacy of immunotherapy in the treatment of melanoma. TCVs represent a promising approach for boosting T cell responses against tumor antigens without significantly aggravating toxicity.

Therapeutic cancer vaccines aiming to mount anti-hTERT immune responses have been evaluated with several platforms, including peptide, mRNA, and DNA-based approaches [28]. UV1 is a multipeptide therapeutic vaccine that has demonstrated HLA-independent induction of vaccine-specific T cell responses in patients treated across three completed phase I/IIa clinical trials [29]. Effective induction of robust T cell responses is a prerequisite for the potential clinical activity of a TCV. While T cell responses after therapeutic vaccination have been well documented in peripheral blood, there is still a need to further elucidate vaccine-specific T cell trafficking after peripheral priming and their interaction with the tumor microenvironment.

Tumor hTERT protein expression was confirmed in all evaluable biopsies using combined hTERT and melanoma cell immunofluorescence staining. The relatively high fraction of hTERT positive melanoma cells (median 72.7%) supports the concept of hTERT being a relevant tumor antigen also in otherwise heterogenous tumors. As hTERT activation serves essential tumorigenic functions, the hTERT negative melanoma cells may be bystander cells contributing less to metastasis and are thus less relevant for clinical progression. The intensity of hTERT staining in melanoma cells was significantly higher in clinical progressors. The increased staining intensity may be related to a higher tumoral hTERT activity, which is a well-described negative prognostic factor [20–24]. Copy number amplification of the hTERT gene is a mechanism of tumor hTERT activation and associates with high tumor hTERT expression [18, 19]. Two biopsies (N02 and N13) were polyploid for the hTERT gene, and interestingly, these two biopsies had the highest hTERT-Sox10/S100 density based on immunofluorescence. Furthermore, patient N02 demonstrated the strongest T cell proliferation response to in vitro peptide stimulation, possibly indicating tumoral boosting of the immune response. Regrettably, we had only one PBMC sample (week 4) for immune response assessment of patient N13, which did not show a positive immune response. We did not observe mutations in the UV1 region of hTERT, either at baseline or post-treatment, which could potentially render the UV1-specific immune response redundant. Inducing immune responses towards epitopes in the hTERT active site theoretically limits tumor immune escape, as mutations in this region could negatively affect telomerase activity and thus impede tumor growth. We observed mutations in ALT-related DAXX and ATRX genes. However, these missense mutations did not induce the ALT phenotype, as hTERT expression was confirmed by immunofluorescence staining of the same biopsies.

Baseline CD4 or CD8 T cell infiltration was not associated with clinical response, and we did not observe a significant influx of TILs in post-treatment samples. The limitations of our study include the small number of patients with evaluable samples, timing of tissue harvesting, and intratumoral heterogeneity. As the median time to clinical response was 30.2 weeks, tumor tissue sampling at weeks 12–15 may be too early to describe clinically relevant T cell infiltration, although TIL influx after ipilimumab treatment of melanoma has been observed after 18 weeks in other studies [46]. We observed a non-significant trend towards tumor CD8 influx with increased peripheral vaccine-specific T cell responses (Fig. 4B). This observation may fit well with the proposed mechanism of action of a therapeutic cancer vaccine, whereby vaccination promotes the infiltration of T cells into the tumor. Nevertheless, this correlation requires further testing in larger cohorts. Increased expression of the IFN-γ gene signature and genes related to T cell activation and cytokine activity was observed in clinically responding patient N07 (Fig. 4 and Additional file 1: Figure S4). Conversely, the two non-responding patients exhibited relatively higher expression of the immune checkpoints CD276 and VTCN1 (B7-H3 and B7-H4), the latter being upregulated post-treatment.

TCR sequencing is emerging as an important tool for characterizing T cell dynamics and tissue trafficking [47]. By sequencing the rearranged TRB locus, we aimed to elucidate how ipilimumab and hTERT vaccination affected the overall TCR repertoire and whether vaccine-enriched TCR clonotypes were detectable in peripheral blood and tumor biopsies. Our strategy for identifying TCRs related to vaccination consisted of paired TCR sequencing of PBMC samples before and after a 10-day in vitro vaccine peptide stimulation. The TCR clonality of the sample did not increase after the 10-day in vitro stimulation, despite exhibiting strong T cell proliferation responses to vaccine-peptide stimulation. These findings support the concept that the long UV1 vaccine peptides contain multiple epitopes eliciting a diverse T cell response in each patient, rather than single vaccine-specific clonotypes. This hypothesis is further supported by previously published data on diversity among immune responder HLA genotypes and the various HLA restrictions and epitope specificities of vaccine-specific T cell clones [29]. Nevertheless, we identified TCR clonotypes that were significantly enriched after in vitro stimulation and subsequently detected them in unstimulated PBMCs and tumor tissue. Alternative strategies, such as peptide-MHC multimer or IFN-γ positivity sorting, may be superior for accurately detecting vaccine-specific T cell clones and validate our current approach in future studies.

The clinical read-out of our study yielded an ORR of 33%, mPFS of 6.7 months, and mOS of 66.3 months. The clinical outcomes of patients enrolled in a phase 4 clinical trial evaluating ipilimumab monotherapy at Norwegian hospitals during the same period as our study were recently published (n = 151) [34], demonstrating an ORR of 9%, mPFS of 2.7 months, and mOS of 12.1 months.

## Conclusions

Although the small sample size and lack of a control arm limits interpretations of clinical efficacy, our study provides support for further clinical evaluation of UV1 vaccination. Anti-telomerase immune responses were established in 91% of patients, and clinical responses were observed in patients with otherwise less favorable baseline genomic, transcriptomic, and tumor microenvironmental features predictive of CPI efficacy. Currently, five randomized phase II clinical trials are evaluating UV1 in combination with various CPIs across multiple indications (NCT05075122, NCT04742075, NCT04382664, NCT04300244, and NCT05344209).

## Supplementary Information

**Additional file 1: Table S1**. Samples selected for TCR sequencing. **Table S2.** Biopsies used for immunofluorescence staining. **Table S3.** Antibodies and amplification reagents used for multiplex fluorescence IHC. **Figure S1.** Comparison of mutations in baseline and post-treatment biopsy. **Figure S2.** Baseline HLA class I and II expression in clinical responders vs. non-responders. **Figure S3. **Vaccine-enriched TCRs. **Figure S4.** Gene expression profiles at baseline vs. post-treatment. **Figure S5.** Gene set enrichment analysis of differentially expressed genes post-treatment.

## Data Availability

Relevant data are provided in the publication and supplementary material. Further details are available on reasonable request. Whole-exome sequencing data are deposited in the European Genome-Phenome Archive (https://ega-archive.org/) under accession number EGAS00001005253. The requests to access the dataset should be directed at espen.ellingsen@ultimovacs.com.

## Abbreviations

CPI: Checkpoint inhibitor
PD-1: Programmed cell death protein 1
PD-L1: Programmed death-ligand 1
IFN-γ: Interferon-γ
TME: Tumor microenvironment
TCV: Therapeutic cancer vaccine
TAA: Tumor-associated antigen
hTERT: Human telomerase reverse transcriptase
HLA: Human leukocyte antigen
CTLA-4: Cytotoxic T-lymphocyte-associated protein 4
Th1: T helper 1
ORR: Objective response rate
OS: Overall survival
PFS: Progression-free survival
RECIST 1.1: Response Evaluation Criteria in Solid Tumors 1.1.
ECOG: Eastern Cooperative Oncology Group
GM-CSF: Granulocyte–macrophage colony-stimulating factor
PBMC: Peripheral blood mononuclear cell
SI: Stimulation index
SEC3: Staphylococcus aureus enterotoxin C3
TRB: T cell receptor beta
CDR3: Complementarity-determining region 3
FFPE: Formalin-fixed and paraffin-embedded
WES: Whole-exome sequencing
TIL: Tumor-infiltrating lymphocyte
NR: Not reached
BOR: Best overall response
CR: Complete response
PR: Partial response
SD: Stable disease
PD: Progressive disease
SNV: Single nucleotide variant
ALT: Alternative lengthening of telomeres
