# Supplementary material for: Characterization of the T cell receptor repertoire and melanoma tumor microenvironment upon combined treatment with ipilimumab and hTERT vaccination
Source: J Transl Med. 2022 Sep 11;20:419. doi: 10.1186/s12967-022-03624-z (PMC9465869; doi:10.1186/s12967-022-03624-z)
Supplement: Supplementary file 1 — Additional file 1: Table S1. Samples selected for TCR sequencing. Table S2. Biopsies used for immunofluorescence staining. Table S3. Antibodies and amplification reagents used for multiplex fluorescence IHC. Figure S1. Comparison of mutations in baseline and post-treatment biopsy. Figure S2. Baseline HLA class I and II expression in clinical responders vs. non-responders. Figure S3. Vaccine-enriched TCRs. Figure S4. Gene expression profiles at baseline vs. post-treatment. Figure S5. Gene set enrichment analysis of differentially expressed genes post-treatment. [file 12967_2022_3624_MOESM1_ESM.docx]

**Additional Table:**

|  | Weeks | | | | | | | | | | | | | |
| --- | --- | --- | --- | --- | --- | --- | --- | --- | --- | --- | --- | --- | --- | --- |
| Patient | 0 | 4 | 7 | 9 | 12 | 14 | 15 | 17 | 18 | 23 | 24 | 24 | 25 | 26 |
| N01 | PBMC | - | Stimulated, PBMC | - | - | - | - | - | - | - | - | - | PBMC | - |
| N02 | PBMC, biopsy | - | Stimulated, PBMC | - | - | biopsy | - | - | PBMC | - | - | - | - | - |
| N03 | PBMC, biopsy | - | - | - | - | - | biopsy | PBMC | - | - | - | - | - | - |
| N04 | PBMC | - | PBMC | - | - | - | - | - | - | - | PBMC | - | - | - |
| N05 | biopsy, PBMC | - | PBMC | - | - | - | - | - | - | - | - | - | - | - |
| N06 | biopsy, PBMC | - | - | PBMC | - | - | PBMC | - | - | - | - | - | - | - |
| N07 | PBMC | - | Stimulated, PBMC | - | - | - | - | - | - | - | PBMC | - | - | - |
| N08 | PBMC | - | - | - | - | - | - | - | - | - | - | - | - | PBMC |
| N09 | PBMC, biopsy | - | Stimulated, PBMC | - | - | - | - | - | - | PBMC | - | - | - | - |
| N11 | biopsy, PBMC | - | - | - | - | - | - | - | - | - | - | PBMC | - | - |
| N13 | biopsy, PBMC | PBMC | - | - | - | - | - | - | - | - | - | - | - | - |
| N14 | PBMC | - | - | - | - | - | - | - | - | - | - | - | - | - |

**Table S1. Samples selected for TCR sequencing**

“Stimulated” refers to PBMC samples after 10-day in vitro vaccine peptide stimulation

**Table S2. Biopsies used for immunofluorescence staining**

| **Patient** | **Baseline sample location** | **Post-treatment sample location** | **Post-treatment timepoint** |
| --- | --- | --- | --- |
| **N01** | Subcutaneous, left thorax | Subcutaneous, left thorax | 12 |
| **N02** | Muscle, proximal right leg | Muscle, proximal right leg | 14 |
| **N03** | Subcutaneous, left side of neck | Subcutaneous, left side of neck | 15 |
| **N04** | Lymph node, central mesenterium | Lymph node, central mesenterium | 12 |
| **N05** | Liver, segment 2 |  |  |
| **N06** | Cutis, left cheek |  |  |
| **N07** | Subcutaneous, left shoulder | Lymph node, left axillae | 13 |
| **N11** | Lymph node, left supraclavicular |  |  |
| **N13** | Tumor deposit, left fossa poplitia |  |  |

**Table S3. Antibodies and amplification reagents used for multiplex fluorescence IHC.**

| **Order** | **Antigen retrieval** | **Marker** | **Clone** | **Host Species** | **Dilution** | **Company** | **Amplification/ enzyme reagent**† | **Fluorophore^#^** |
| --- | --- | --- | --- | --- | --- | --- | --- | --- |
| 3. | pH6 | CD4 | ERP6855 | Rabbit | 1:80 | Abcam | Opal HRP | Opal 570 |
| 7. | pH6 | CD8a | C8/144B | Mouse | 1:100 | Invitrogen | ImPress | Opal 690 |
| 5. | pH6 | PD-L1 | E1L3N | Rabbit | ready | Akoya | ImPress | Opal 540 |
| 6. | pH6 | TERT | Ab230527 | Rabbit | 1:400 | Abcam | ImPress | Opal 620 |
| 8. | pH6 | Sox10/  S100 | EP268-1/  4C4.9 | Rabbit/Mouse | Ready (mix 1:1) | Akoya | Opal HRP | Opal 780 |
| 9. | - | DAPI | - | - | - | Akoya | - | Akoya |

The staining procedure included 8 cycles of antigen retrieval, incubation with primary antibody, amplification system and fluorophore labeling. The final cycle was performed by DAPI staining. Antigen retrieval was performed at 95 °C, 15min. †Amplification systems ImmPRESS® HRP or Opal HRP or SignalStain® Boost IHC Detection Reagent (HRP, Goat) were used: The ImmPRESS® HRP Anti-Mouse IgG (Peroxidase) (Cat. No: MP-7402-50) and Anti-Rabbit IgG (Peroxidase) Polymer Detection Kits, made in Horse (Cat No: MP-7401-50) (Vector Laboratories) and SignalStain® Boost IHC Detection Reagent (HRP, Goat) (#63707 CellSignaling); Opal™ Polymer anti-Rabbit+anti-Mouse HRP Kit (Cat No: ARH1001EA) (Akoya). #Florescent labeling was performed by Opal fluorophores at dilution 1:100, 15 min.

**Additional Figures:**

**Figure S1. Comparison of mutations in baseline and post-treatment biopsy**

**Figure S2. Baseline HLA class I and II expression in clinical responders vs. non-responders**


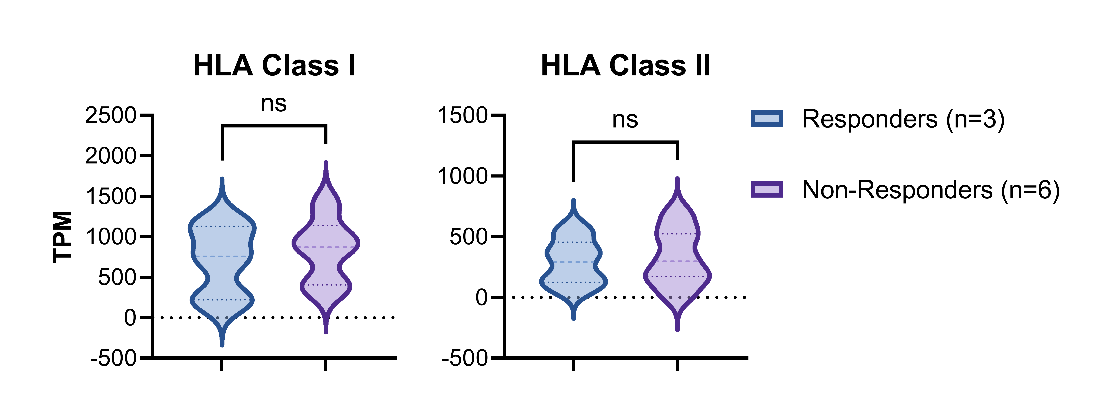


**Figure S3. Vaccine-enriched TCRs**


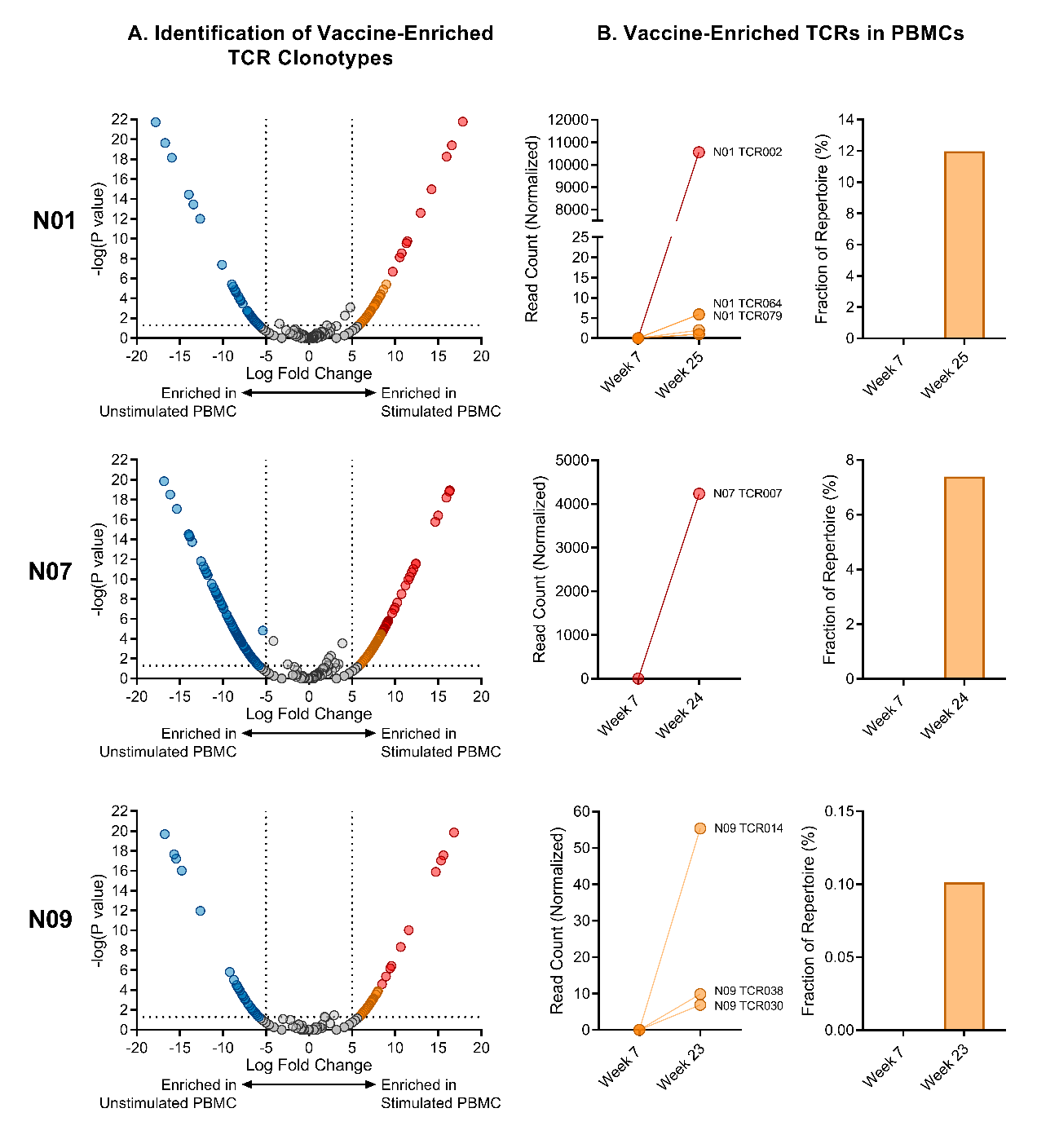


**Figure S4. Gene expression profiles at baseline vs. post-treatment**


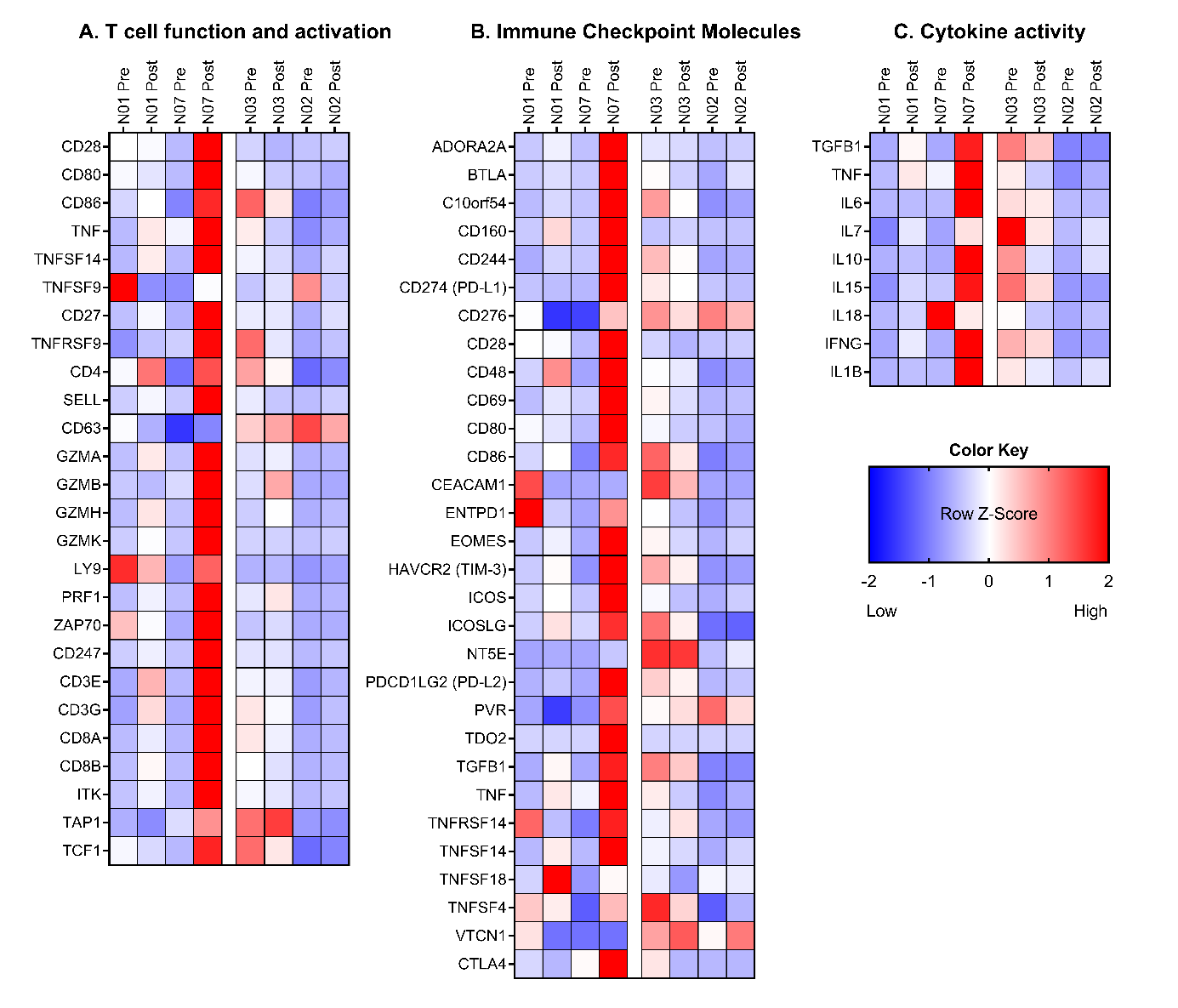


**Figure S5. Gene set enrichment analysis of differentially expressed genes post-treatment**


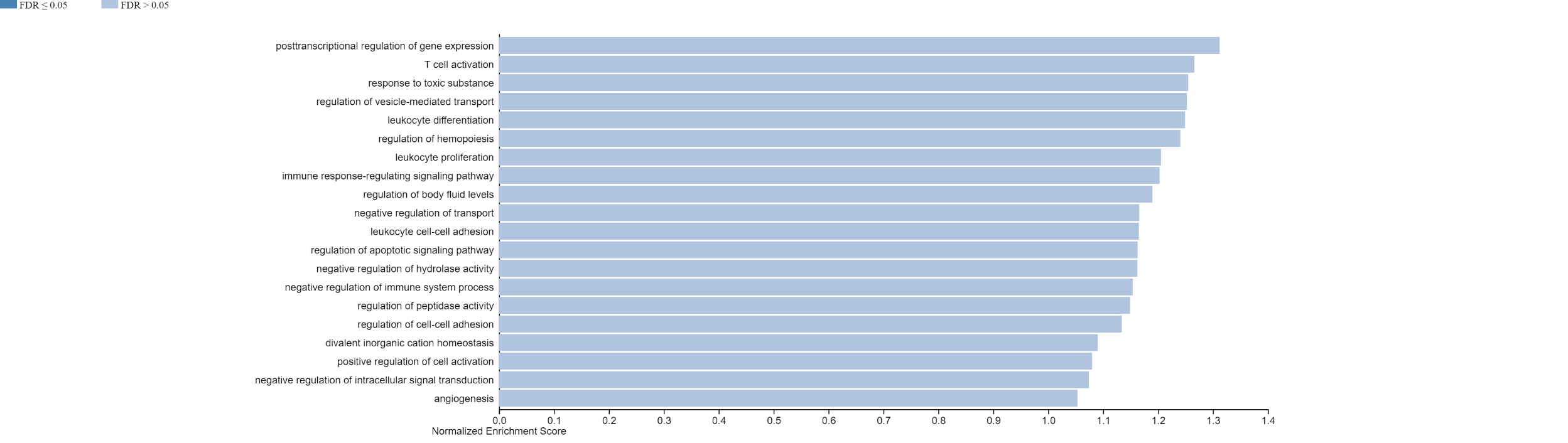
**N01**

**
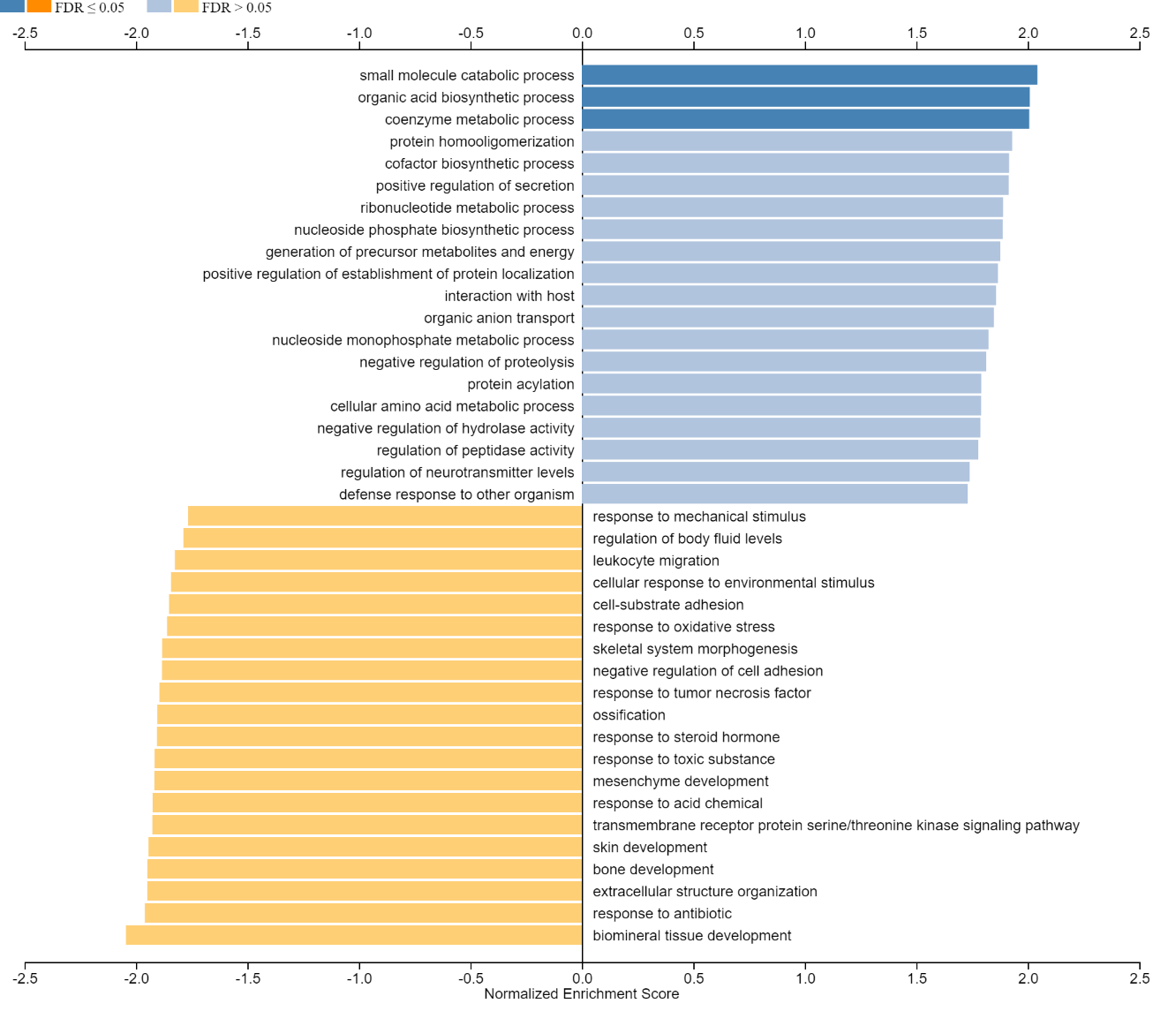
N02**
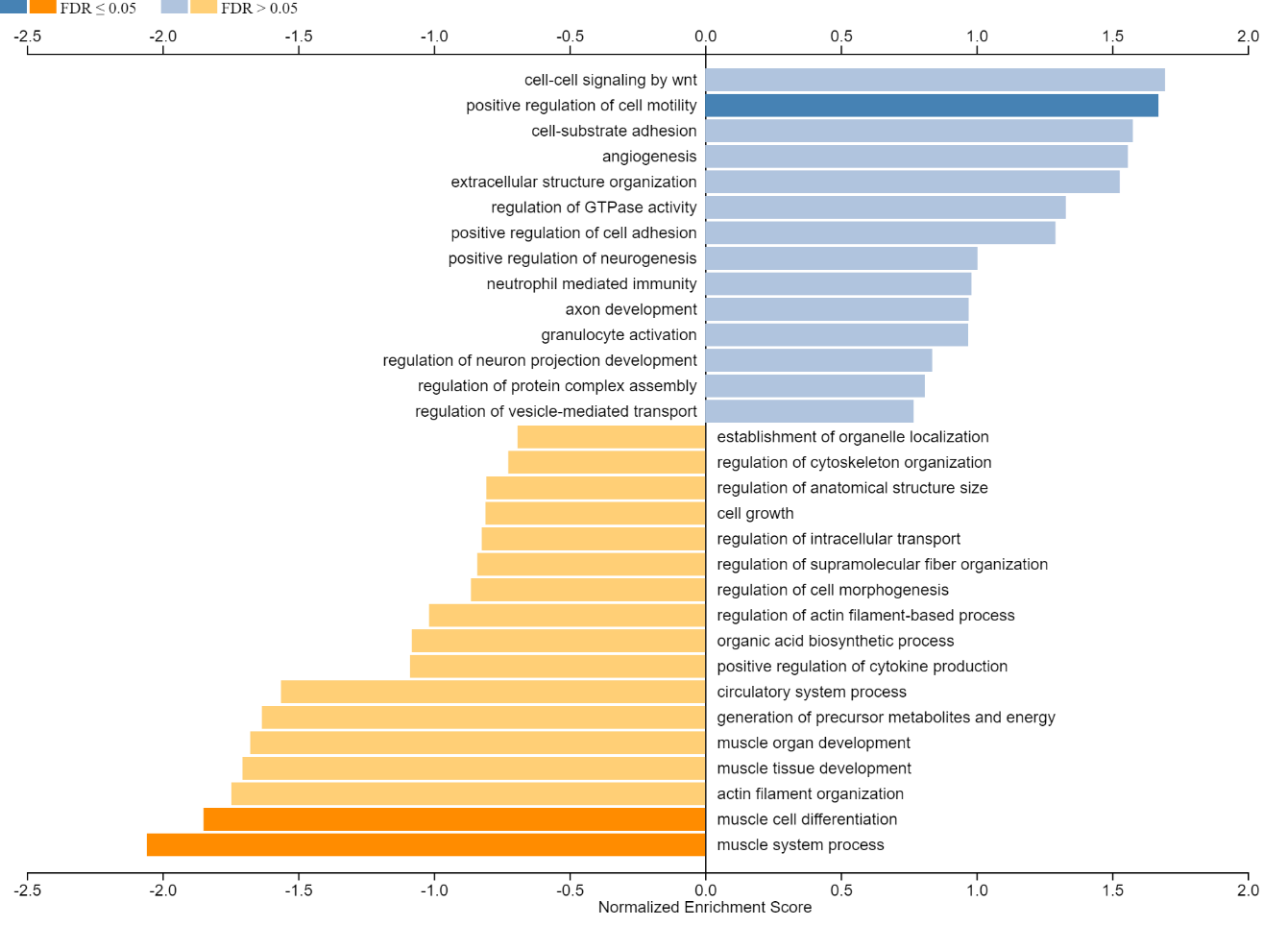


**N03**

**N07**

**
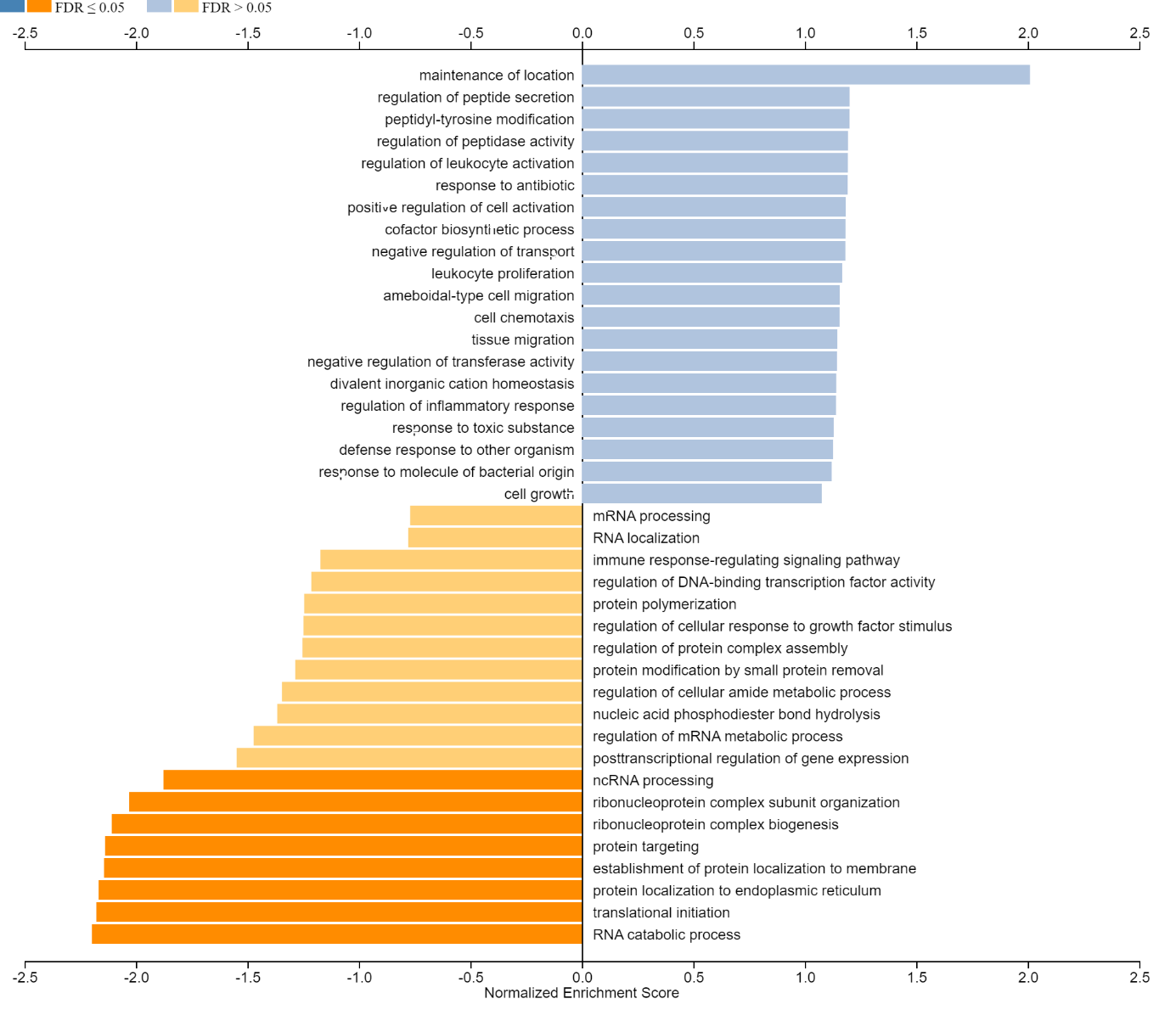
**

**Additional Methods:**

**Normalization**

Count normalization was performed by downsampling. The sampling depth was chosen to be 99% of the total count in the smallest sample in the set. The sampling depth determined how many times a single clone is drawn from each sample. This ensured that all normalized samples have the same total clone count, and clones were represented according their original frequency. To ensure fair representation of small clones, this downsampling was performed 100 times and the average subsampled count was taken for plotting.

**Differential abundance**

Identifying significantly expanded clones from repertoire sequencing data counts is a differential abundance calculation, similar to differential expression analyses. Our tool of choice was based on the fact that replicates were not available in our dataset. EdgeR provides a differential expression procedure to compare only two samples^36^.

There are two steps in the differential abundance procedure: 1) total count normalization and 2) a statistical test for the difference in normalized counts between the two samples. The normalization applied was downsampling as described above. The statistical test was the exact test implemented in the edgeR package, which is an exact binomial test (*exactTest*) generalized to overdispersed counts. To assess the robustness of the results, the exact test was performed on 100 different downsamples of the original samples, and clones were sorted by their average log fold change and average rank. Significantly enriched TCR clonotypes were named according to their rank in terms of log fold change. TCR clonotypes present at baseline were filtered out before searching for the vaccine-enriched TCRs in unstimulated PBMCs and biopsy.
